# Hydroxychloroquine plus azithromycin and early hospital admission are beneficial in COVID-19 patients: Turkish experience with real-life data

**DOI:** 10.3906/sag-2005-82

**Published:** 2021-02-26

**Authors:** Elif TANRIVERDİ, Mustafa ÇÖRTÜK, Binnaz Zeynep YILDIRIM, Efsun Gonca UĞUR CHOUSEIN, Demet TURAN, Halit ÇINARKA, Mehmet Akif ÖZGÜL, Erdoğan ÇETİNKAYA

**Affiliations:** 1 Department of Pulmonology, University of Health Sciences Yedikule Pulmonary Diseases andThoracic Surgery Education and Research Hospital, İstanbul Turkey

**Keywords:** COVID-19, pneumonia, azithromycin, hydroxychloroquine, treatment, mortality, biomarkers

## Abstract

**Background/aim:**

New treatment regimens for COVID-19, which has threatened the world recently, continue to be investigated. Although some of the treatments are promising, it is thought to be early to state that there is definitive treatment. Experiences and treatment protocol studies from treatment centers are still important. The aim of this study is to evaluate factors affecting the treatment process of the first cases followed in our clinic.

**Materials and methods:**

The consecutive hospitalized patients with COVID-19 pneumonia were analyzed in this retrospective and cross-sectional study. Data were recorded from the electronic and written files of patients.

**Results:**

Eighty-three patients were evaluated. The median age was 50 ± 15 years. Forty-eight (57.8%) patients had one or more comorbidities. The most common comorbidity was hypertension. The most common symptom was cough in 58 patients (70%). The overall mortality was 15%, and 85% of the patients were discharged. The time between the onset of symptoms and hospitalization was statistically significantly longer in deceased patients (P = 0.039). Age, D-Dimer, troponin, CK, CK-MB, ferritin, procalcitonin, and neutrophil to lymphocyte ratio were statistically significantly higher in deceased patients than survivor patients. In subgroup analysis, in the patients receiving azithromycin plus hydroxychloroquine and other antibiotics plus hydroxychloroquine, the duration of hospitalization was shorter in the azithromycin group (P = 0.027).

**Conclusion:**

Early treatment and early admission to the hospital can be crucial for the better treatment process. Combination therapy with azithromycin may be preferred in the first treatment choice because it can shorten the length of hospital stay.

## 1. Introduction

In December 2019, the Hubei Province in Wuhan, China reported a pneumonia epidemic of unknown etiology. A new coronavirus known as severe acute respiratory syndrome coronavirus 2 (SARS-CoV-2) was detected in the throat swab samples of affected patients, and the disease caused by this virus was named as coronavirus disease 2019 (COVID-19) [1]. As of the first week of March 2020, cases of COVID-19 started to emerge in Turkey.

Because of the absence of precise management protocols, new treatment regimens are being investigated for the management of COVID-19. Some of these treatments have delivered unfavorable outcomes, whereas some of them have provided promising results. Although treatment results are being meticulously presented in clinical trials, it is considered too early to clearly state the accuracy of the treatment methods [2]. For this reason, experiences in the treatment processes coming from treatment centers and studies of treatment protocols are still crucial.

In our clinic, which is a tertiary chest diseases training and research hospital, we have been treating patients in accordance with the COVID-19 scientific board recommendations created by the Turkish Ministry of Health since the beginning of pandemic. Therefore, the objective of this study is to evaluate the demographic data, mortality rates, and factors affecting mortality and treatment processes in the first cases followed up in our clinic.

## 2. Material and methods

In this retrospective and cross-sectional study, we retrospectively analyzed the consecutive hospitalized patients with COVID-19 pneumonia between March 13, 2020 and April 15, 2020. This study was conducted in a tertiary chest diseases training and research hospital that has been chosen as a pandemic hospital. We recorded age, sex, contact history, smoking history, and comorbidities from the electronic and written files of patients. We also recorded complete blood count, blood chemical analysis, coagulation testing, liver and renal function tests, electrolytes, C-reactive protein, procalcitonin, lactate dehydrogenase, D-Dimer, plasma fibrinogen, and creatine kinase (CK). In addition, we evaluated the symptoms of patients, the time from the onset of symptoms to hospitalization, the need of oxygen inhalation therapy during hospitalization, noninvasive mechanical ventilation (NIMV), and intensive care unit (ICU) needs and treatment regimens. Radiological findings were recorded by evaluating the thickness of 3 mm axial images with 16-channel multidetector chest computed tomography (CT). Patients were defined as possible and confirmed cases according to the case definitions published by the Ministry of Health [3].

Possible cases comprise:

A: At least one of the signs and symptoms of fever or acute respiratory disease (cough and respiratory distress), AND

• The clinical picture cannot be explained by another cause/disease AND

• Within 14 days before the onset of symptoms, patient and relatives’ history of being abroad 

B: At least one of the signs and symptoms of fever or acute respiratory disease (cough and respiratory distress), AND

• Close contact with confirmed COVID-19 case within 14 days prior to the onset of symptoms 

C: At least one of the signs and symptoms of fever and severe acute respiratory infection (cough and respiratory distress), AND

• The need for hospitalization (SARI*) AND

• The clinical picture cannot be explained by another cause/disease

*SARI (severe acute respiratory infections: fever, cough in a patient with acute respiratory infection that developed in the last 14 days and dyspnea, tachypnea, hypoxemia, hypotension, and the need for hospitalization due to changes in diffuse radiological signs and consciousness)

D: Cough or shortness of breath with a sudden onset of fever and no runny nose

Confirmed cases comprise: 

Detection of SARS-CoV-2 in a molecular method in one of the cases that fits the possible case definition

Oxygen saturation in room air <90%, respiratory rates ≥30 / min, widespread pneumonic infiltrations on chest X-rays and/or thorax computed tomography (CT), or detection of acute organ dysfunction were defined as “severe disease”. 

Hydroxychloroquine 200 mg tablets (2 tablets twice a day for the first day; 1 tablet twice a day for the following 4 days) were administered to every inpatient with COVID-19 pneumonia from the first day of hospitalization. Third-generation cephalosporin and quinolone were added as an empiric antibiotic for all patients. The scientific committee, determined by the Ministry of Health, regularly updated the management of COVID-19. In addition to hydroxychloroquine, azithromycin treatment, depending on the decision of the treating physician, was recommended on March 25 [3]. From this date, azithromycin tablets (500 mg per day for the first day; 250 mg per day for the following 4 days) plus hydroxychloroquine were preferred for the patients of our clinic. With the proposal of the scientific committee, favipiravir 200 mg tablets (1600 mg loading dose twice a day; 600 mg twice for the following 4 days) or lopinavir 200 mg/ritonavir 50 mg tablet (2 tablets twice a day for 10–14 days) were mostly preferred in patients with oxygen saturation <90% on room air, respiration rate over 30 breaths at rest, and rapid clinical and radiological progression of the disease.

Electrocardiography was performed on all patients and an electrocardiogram was evaluated before treatment was started.

Patients who are still undergoing inpatient treatment, patients with positive influenza test results, and patients diagnosed with bacterial infection were excluded from the study.

 The ethics committee of our institution approved the protocol of this study.

### 2.1. Statistical analysis

We presented continuous measurements as mean ± standard deviation if they were normally distributed or median (with minimum and maximum). However, if continuous measurements are not normally distributed, then we presented the categorical variables as count (%). For comparing the groups showing normal distribution, we used an independent t-test. We used the Mann–Whitney U test for continuous variables showing the nonnormality of distribution. Moreover, we used the chi-square test for testing the relationships between categorical variables. To evaluate the relationship between symptom duration until hospital admission and mortality, we determine a cut-off value for symptom duration by ROC analysis. A P-value of <0.05 was considered statistically significant in this study.

## 3. Results

In this study, 83 patients, of whom 41 (49%) were confirmed cases and 42 (51%) were probable cases, were evaluated. Sixty (72.2%) patients were male. The median age of patients was 50 ± 15 years. Fifteen (18%) patients were smokers, 52 (63%) patients were nonsmokers, and 16 (19%) patients were former smokers. In total, 48 (57.8%) patients had one or more comorbidities. The most common comorbidities were hypertension in 25 (30%) patients, diabetes mellitus in 12 (14%), coronary heart disease in 10 (12%), and asthma in 8 (10%) patients. The most common symptom was cough in 58 patients (70%) and fever in 44 (53%) patients, followed by dyspnea in 43 (51%) patients, fatigue in 30 (36%) patients, myalgia in 20 (24%) patients, gastrointestinal symptoms including nausea, diarrhea, and loss of appetite in 20 (24%) patients, sputum in 10 (12%) patients, sore throat in 7 (8%) patients, headache in 6 (7%) patients, and hemoptysis in 2 (2.5%) patients. Symptoms began at a median of 7 days (min: 1–max: 30) before hospitalization.

The most common radiological localizations were observed in the right lower (84%) and left lower (80%) lobes, respectively. Bilateral ground-glass opacity (72%) was the most common radiological finding on chest CT. Table 1 presents the radiological features of the patients. Of 83 patients, 68 (82%) had bilateral involvement and 15 (18%) had unilateral involvement.

**Table 1:** Radiological features in patients.

	Patient number (%)
Lung localization	
-Right upper lobe	63 (76)
-Right middle lobe	54 (65)
-Right lower lobe	70 (84)
-Left upper lobe	57 (68)
-Left lower lobe	67 (80)
Distribution	
-Central	54 (65)
-Peripheral	76 (91)
-Focal or multifocal	59 (71)
-Diffuse	12 (14)
Image findings	
- Bilateral GGO	60 (72)
- Unilateral GGO	12 (14)
-Air bronchogram	28 (33)
-Bronchial vascular enlargement	26 (31)
-Subpleural scarring	19 (23)
-Halo/reverse halo sign	2/20 (2.5/ 25)
-Nodule	8 (9.5)
-Pleural effusion	5 (6)
-Crazy paving	3 (3.6)
-Mediastinal lymph node	2 (2.5)
-Pericardial effusion	2 (2.5)
-Spontaneous pneumothorax	1 (%1.2)

*GGO: ground glass opacity.

There were 38 (46%) severe cases and 45 (54%) mild/moderate cases. In total, 61 (73%) of the patients needed oxygen inhalation therapy with nasal cannula or face mask, 16 (19%) patients were administered NIMV in the hospital ward, and 19 (22%) patients were administered invasive mechanical ventilation (IMV) in the ICU. Average length of stay in ICU was 7 days (min: 1–max: 23). Seven of the 19 patients who were followed up in the ICU were successfully weaned and discharged. One patient died in the hospital ward. The overall mortality was 15%, and 85% of the patients were discharged. The median length of hospital stay was 8 days (min: 1–max: 29). Unexpected arrhythmia or cardiac events were not observed during hospitalization period. 

The comparison of clinical and laboratory parameters of survivor and deceased patients revealed that the time between the onset of symptoms and hospitalization was statistically significantly longer in the deceased patients. Mortality was statistically significantly higher if the time from the onset of symptoms to the hospital admission was more than 9 days (≤9 days vs >9 days, P = 0.042, AUC: 2.679, sensitivity: 54%, specificity: 74%, positive predictive value: 28%, negative predictive value: 90%, and accuracy: 71.1%). Age, D-Dimer, troponin, CK, CK-MB, ferritin, procalcitonin, and neutrophil to lymphocyte ratio (NLR) were statistically significantly higher in deceased patients than the survivor patients. The lymphocyte count was lower in deceased patients. However, there was no statistically significant difference in terms of the duration of hospitalization between survivor and deceased patients (Table 2).

**Table 2:** Comparison of clinical and laboratory parameters of nonsurvivors and survivors.

	SURVIVOR (n = 70)	NONSURVIVOR (n = 13)	P
Age (years)	47.7 ± 15	66.8 ± 9.4	<0.001
Presence of comorbidity (n, %)	37 (52.8%)	11 (%84.6)	0.033
Symptom duration before hospitalization (day)	7.4 ± 4.6	11.4 ± 7.3	0.039
Duration of hospitalization (day)	9.5 ± 4.4	7.5 ± 4.5	0.09
Sedimentation rate (mm/hour)	59.7 ± 35.8	60.7 ± 42.8	0.945
D- Dimer (mg/L)	1.35 ± 3.19	1.4 ± 1.17	0.007
Troponin (pg/mL)	3.8 (0.6–67.9)	14.6 (3–7620)	<0.001
CK (U/L)	114 (21–1065)	239.5 (44–1862)	0.039
CK-MB (U/L)	22.55 ± 13.11	36.25 ± 8.14	<0.001
Ferritin (ng/mL)	180 (2–1500)	571.9 (277–1500)	0.008
Fibrinogen (mg/dL)	436.6 ± 128.5	503.75 ± 131.6	0.178
Procalcitonin (mg/L)	0.04 (0.01–1.4)	0.34 (0.02–38.8)	0.001
WBC (10e3/uL)	8.35 ± 3.6	9.32 ± 4.96	0.716
Monocyte (10e3/uL)	0.67 ± 0.39	0.52 ± 0.34	0.092
Eozinophyl (10e3/uL)	0.08 ± 0.13	0.02 ± 0.04	0.095
Lymphocyte (10e3/uL)	1.5 ± 0.73	0.83 ± 0.6	<0.001
Neutrophyl (10e3/uL)	6.03 ± 3.41	7.93 ± 5.05	0.257
NLR (10e3/uL)	4.98 ± 4.5	18.14 ± 23.6	0.006

*WBC: white blood cell; NLR: neutrophil to lymphocyte ratio.

Of all patients, 18 received lopinavir/ritonavir and 9 received favipiravir therapy. Five of the patients who took these drugs died. None of them had received azithromycin for initiation therapy. No patient received thrombolytic therapy or immunoglobulin therapy.

We performed subgroup analysis in the patients receiving azithromycin plus hydroxychloroquine, and other antibiotics plus hydroxychloroquine, by excluding patients who were administered favipiravir and lopinavir/ritonavir. There was no statistically significant difference between the two groups in terms of age, sex, the time between the onset of symptoms, D-Dimer, troponin, CK, CK-MB, ferritin, procalcitonin, and NLR. Additionally, there was no statistically significant difference between the two groups in terms of need for ICU and mortality. However, the duration of hospitalization was shorter in the group receiving azithromycin than the group receiving other antibiotics (Table 3).

**Table 3:** Comparison of treatment group with and without azithromycin.

	Azithromycin plus hydroxychloroquine group (n = 26)	Other antibiotics plus hydroxychloroquinegroup (n = 30)	P
Age (years)	51.65 ± 13.41	46.30 ± 17.07	0.203
Sex (male/female)	18/8	21/9	0.950
Presence of comorbidity (n, %)	16 (61.5 %)	15 (%50)	0.386
Symptom duration before hospitalization (day)	7.96 ± 5.28	7.93 ± 5.42	0.605
Duration of hospitalization (day)	6.68 ± 2.23	8.16 ± 2.56	0.027
Need of ICU (n, %)	5 (19)	6 (20)	0.942
Mortality (n, %)	5 (19)	3 (10)	0.324
Troponin (pg/mL)	4.35 (0.6–113.9)	3.6 (1.5–7620)	0.667
CK (U/L)	121.5 (21–1862)	101 (23–760)	0.782
CK-MB (U/L)	20.20 (7.5–51.70)	19.99 (10.5–98.3)	0.952
D-Dimer (mg/L)	0.62 (0.27 ± 11.47)	0.53 (0.18–6.99)	0.393
Ferritin (ng/mL)	299.5 (37–1500)	155.1 (2–1474)	0.243
Procalcitonin (mg/L)	0.04 (0.01–1.4)	0.04 (0.01–38.86)	0.904
Lymphocyte (10e3/uL)	1.37 (0.32–4.18)	1.39 (0.2–3.71)	0.657
NLR (10e3/uL)	3.05 (0.90–33.69)	4.17 (1.21–87.75)	0.301

*ICU: intensive care unit; CK: creatine kinase; NLR: neutrophil to lymphocyte ratio.

## 4. Discussion

COVID-19 is a pandemic that still lacks a definitive and approved treatment method. For this reason, it is important to share the predictors and different treatment approaches from real-life data to reduce mortality and morbidity. Our study demonstrated that early hospital admission reduces hospital stay and mortality. To the best of our knowledge, this is the first report on this issue. In addition, it has been seen that the combination of azithromycin and hydroxychloroquine treatment can be effective in reducing the length of hospital stay.

Death rates reported in the literature vary from 1% to more than 7% in the COVID-19 pandemic. Overall case mortality in large scans where the denominator has many mild or asymptomatic cases across the entire population were reported as less than 1%, but in the studies with a much smaller denominator where the hospitalized patients were evaluated, case death rates were reported above 5% [4]. Kim et al. did not observe mortality in their study, which reported the first cases from Korea. The authors attributed these results to the low number of elderly and patients with comorbidities. In addition, some of the patients were asymptomatic in the prodromal period detected by surveillance test after exposure to virus [5]. However, mortality rates varied between 13% and 16% in some studies [6–8]. The mortality was 15% in our clinic. Our data included the early times of the epidemic, and a relatively short time frame of the COVID-19 pandemic. Also, this is a single-center study. Further studies including a multicenter cohort and longer time of pandemic are needed. In our patients with mortality, in accordance with the literature, the presence of comorbidities, NLR, age, cardiac enzymes, D-dimer, procalcitonin and ferritin were higher in the deceased group than survivor [9–11]. In addition to the literature, the time from the onset of symptom to hospitalization was longer in the deceased group than in the survivors.

From the beginning of the COVID pandemic, we believed that the patients’ early hospital admission and early treatment can be beneficial. Indeed, our study results supported us with lower mortality among the patients who were admitted earlier. Patients with late admission were mostly our first patients. With the appearance of first patients, taking fast precautions and establishing public spots related to the symptoms and signs of the disease rapidly increased the patients’ awareness. Early hospital admission after the onset of symptoms appears to be effective on mortality. To the best our knowledge, this is the first report regarding this issue.

Effective and safe treatment strategies still need to be evaluated to treat the patients before the development of irreversible respiratory complications, to reduce the infectious process, and to prevent disease spread. Hydroxychloroquine treatment was administered to all the patients as soon as they were hospitalized without waiting for PCR results. In addition, in the first cases, third-generation cephalosporin and quinolone groups were employed for empirical treatment. Later, azithromycin was added in line with the Turkish scientific committee and recommendation of the literature.

Hydroxychloroquine accumulates in the cellular organelles creating acidic environment to inhibit the replication of different viruses by interfering with endosome/lysosome trafficking or viral protein maturation during virions maturation. Azithromycin acts as an acidotropic lipophilic weak base which modulate the pH of endosomes and trans-Golgi network, it has a similar mechanism of action with hydroxychloroquine [12]. A recently published study reported that it may be beneficial to combine azithromycin and hydroxychloroquine in the early reduction of contagiousness and in the treatment of COVID-19 [13]. However, right after that, another study was published that claimed contrariwise. The authors of that study stated they could not find any evidence showing that this combination therapy provided a strong clinical response in severe patients who were hospitalized [14]. In our study including real-life data, we found that the use of azithromycin in combination with hydroxychloroquine in a patient group requiring hospitalization may reduce length of hospital stay, independently from the factors of comorbidity, age, and sex. This result supported the study by Gautret et al. [13]. Early preventive measures are essential to manage this pandemic situation. However, after contracting the disease, the discharge of patient with successful outcome needs to be expedited to effectively use the hospital’s capacity. Therefore, the length of hospital stay is critical. The organization of all the factors that positively affect this situation will enable us to manage the pandemic process in the best way. Therefore, we believe that azithromycin may be considered in the treatment of COVID-19 pneumonia.

The most important limitation of our study is the relatively small sample size. In this study, it was observed that various risk factors may influence mortality in COVID-19 pneumonia. More patient groups are required to evaluate whether these risk factors are independent risk factors.

In conclusion, early treatment and early admission to the hospital can be crucial for the better treatment process. Combination therapy with azithromycin may be preferred in the first treatment choice because it may shorten the length of hospital stay. Age, presence of comorbidity, increased cardiac markers, increased D-Dimer, increased NLR, high procalcitonin, and ferritin are some factors that may also be used to predict mortality.

## Author contributions

All authors have read and agreed with the content of the manuscript. Each author has participated sufficiently in the work to take public responsibility for appropriate portions of the content. 

## Ethical approval

Ethics committee approval was received for this study from Hamidiye Scientific Research Ethics Committee of University of Health Sciences Turkey (2020/3/54). Consent form from patients was not received because of being a retrospective study. 
